# Early Diagnosis and Follow-Up of a Novel Homozygous Mutation in *SOST* Gene in a Child with Recurrent Facial Palsy: A Case Report and Review of the Literature

**DOI:** 10.3390/ijms26178175

**Published:** 2025-08-22

**Authors:** Fabio Acquaviva, Giorgia Bruno, Federica Palladino, Alfonso Rubino, Carmela Russo, Maria Pandolfi, Eugenio Maria Covelli, Eloisa Evangelista, Luigia De Falco, Alfonsina Tirozzi, Daniele De Brasi, Antonio Varone

**Affiliations:** 1 Medical Genetics Unit, Department of General and Emergency Pediatrics, AORN Santobono-Pausilipon, 80122 Naples, Italy; f.acquaviva@santobonopausilipon.it (F.A.); d.debrasi@santobonopausilipon.it (D.D.B.); 2 Pediatric Neurology Unit, Department of Neurosciences, Santobono-Pausilipon Children’s Hospital, 80129 Naples, Italy; 3 Pediatric Neuroradiology Unit, Department of Neurosciences, Santobono-Pausilipon Children’s Hospital, 80131 Naples, Italy; 4 AMES, Centro Polidiagnostico Strumentale, srl, 80013 Naples, Italy

**Keywords:** *SOST*, SOST-related sclerosing bone dysplasia, recurrent facial palsy

## Abstract

Recurrent facial palsy is a rare event in the pediatric population, mostly idiopathic or associated with common comorbidities or, rarely, observed in syndromic conditions. However, some cases are difficult to explain and need more accurate diagnostic approaches. In this work, we describe a pediatric case of recurrent facial palsy secondary to hyperostosis of the skull and narrowing of the neural foramina related to a SOST-related sclerosing bone dysplasia. To our knowledge, this is the first Italian case that is also related to a novel loss-of-function variant in the *SOST* gene. We highlight the clinical relevance of a proper early diagnosis and the need for correct monitoring of the clinical evolution, considering the natural history of the disease, to prevent/reduce severe neurological complications.

## 1. Introduction

*SOST* gene (17q12-q21) encodes for sclerostin, a 213-amino acid-long secreted glycoprotein of the DAN family, which is highly conserved across vertebrate species. *SOST* is expressed in several tissues, including bone, cartilage, kidney, liver, heart, lung, pancreas, and skeletal muscle [1]. In bone, sclerostin is primarily secreted by osteocytes with osteoblasts and osteoclasts that also express *SOST* at lower levels [1]. Furthermore, sclerostin protein expression is also detected in several other tissues, including mineralized hypertrophic chondrocytes, odontoblasts, and cementocytes [1]. Sclerostin is a negative regulator of bone formation [2] and inhibits the Wnt/β-catenin signaling pathway and the osteoblastic bone formation [3]. As a consequence, sclerostin deficiency results in osteoblast hyperactivation and bone overgrowth.

Sclerostin defects are responsible for the metabolic bone disorders known as SOST-related sclerosing bone dysplasia, which include three different entities: Sclerosteosis (MIM 269500), Van Buchem Disease (VBD; MIM 239100), and the SOST-related craniodiaphyseal dysplasia (CDD; MIM 122860) [4].

Sclerosteosis is caused by biallelic pathogenic variants in *SOST* [5], which result in the loss of a functional C-terminal domain necessary for the secretion of sclerostin protein from the endoplasmic reticulum [6]. Patients with Sclerosteosis display overgrowth of the skull bones, mandible, clavicles, ribs, pelvis, and long bones, and are often exceptionally tall and have excessive body weight due to high skeletal weight. Hand abnormalities, including cutaneous syndactyly, brachyphalangy, and nail dysplasia, are described in at least one third of patients [7].

Van Buchem Disease (VBD, also known as hyperostosis corticalis generalisata or endosteal hyperostosis) shows similar manifestations to those of Sclerosteosis, including increased thickening of the jawbone, skull, ribs, and long bones, but, in general, they appear as less severe compared to those observed in the Sclerosteosis phenotype. Bone overgrowth in VBD results in very high bone mineral density (BMD) that can lead to facial distortion accompanied by deafness and facial palsy due to bone entrapment of cranial nerves [8]. Otherwise, in Van Buchen Disease, normal stature and no hand abnormalities are found [9]. This “milder” phenotype reflects the reduced rather than abolished *SOST* expression related to the VBD-specific biallelic 52-kb deletion downstream *SOST*, which removes a specific regulatory element [10,11].

Both Sclerosteosis and VBD are inherited in an autosomal recessive manner.

Conversely, the SOST-related craniodiaphyseal dysplasia (CDD) is an autosomal dominant condition reported in just two children carrying heterozygous *SOST* pathogenic variants in specific amino acid residues (c.61G>A, Val21Met and c.61G>T; Val21Leu) [12,13]. These variants are located in the secretion signal of *SOST* and prevent the sclerostin secretion, possibly acting via a dominant negative mechanism [12]. Patients show progressive overgrowth of the craniofacial bones, deafness, facial palsy, and visual disturbance as a result of nerve entrapment [12,13].

Here, we report a pediatric case of very early-onset bilateral recurrent facial palsy secondary to hyperostosis of the skull and narrowing of the neural foramina in a 4-year-old patient. By whole exome sequencing (WES), we identified a novel homozygous c.71delA (p.Gln24ArgfsTer33) variant in the *SOST* gene, which results in a premature stop codon and truncated protein formation.

## 2. Results

### 2.1. Case Report

**Figure 1:**
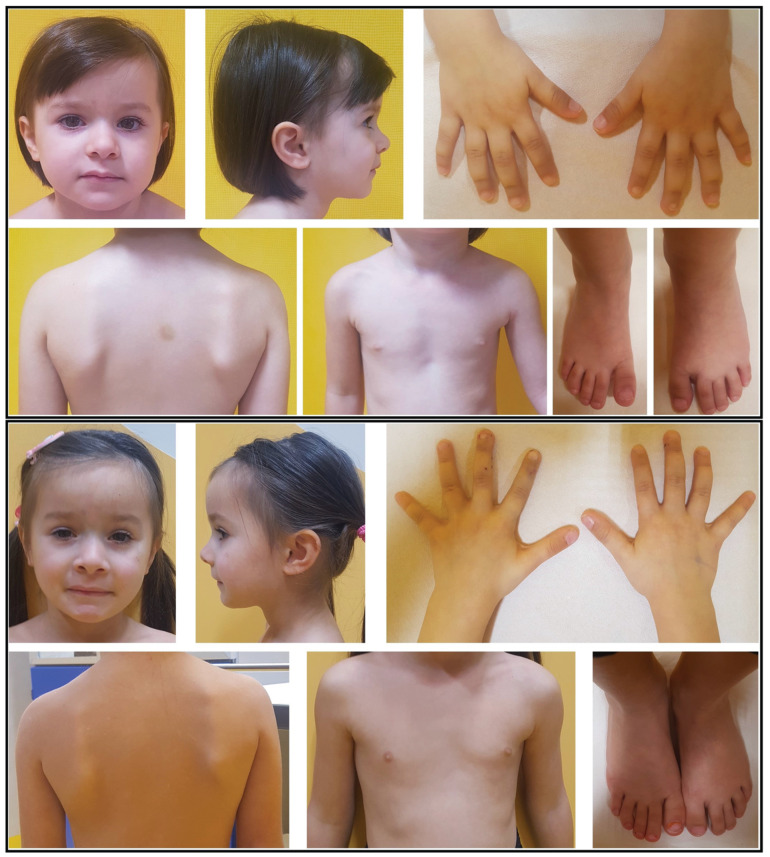
Proband at 3 (**upper** panel) and 4 years (**lower** panel). See also Supplementary Table S1.

An 18-month-old female, second child of healthy, unrelated Italian parents, was initially admitted to the Neurology Outpatient Service for bilateral recurrent facial nerve palsy (Figure 1). Pre- and perinatal anamnesis is unremarkable.

**Figure 2:**
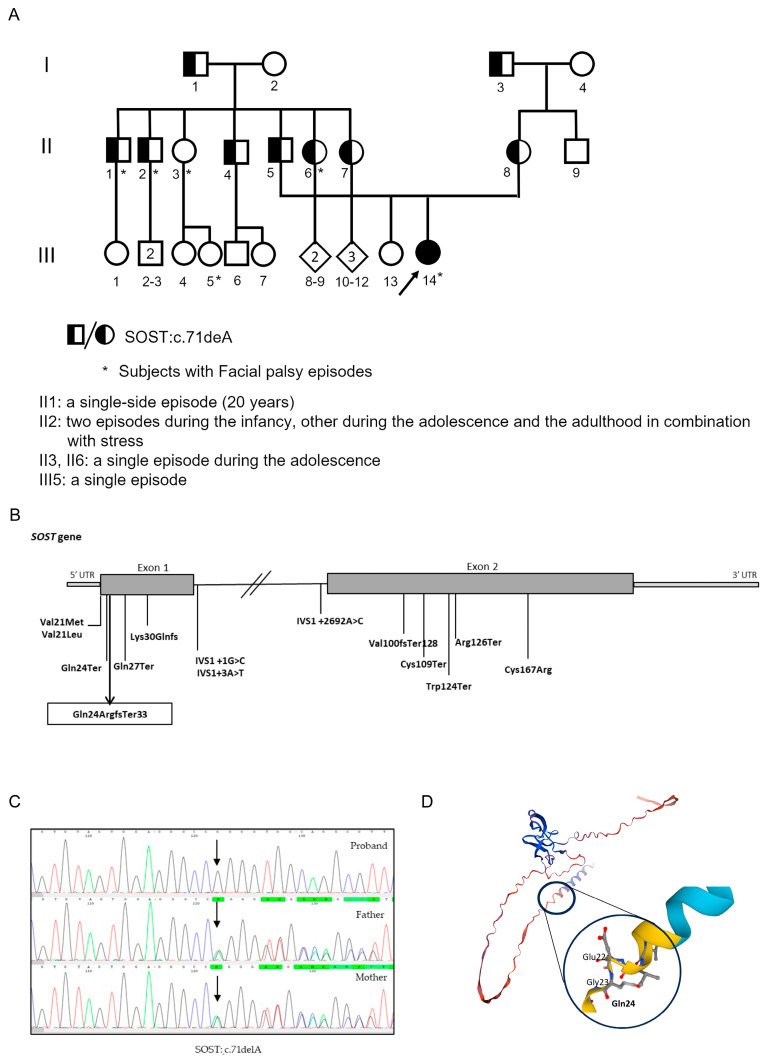
(**A**). Three-generation pedigree. Filled black symbols indicate the individuals affected with Sclerosteosis, and the shapes partially filled indicate the carriers with heterozygous mutation. The proband is marked with the arrow. (**B**). Schematic representation of Sclerosteosis-relevant mutations reported ClinVar pathogenic mutations in *SOST* gene (see also Supplementary Table S2). The new mutations are boxed in the rectangle. (**C**). Sanger sequencing results of *SOST* gene variant c.71delA (p.Gln24ArgfsTer33) in the proband (homozygous) and her heterozygous parents. The arrow indicates the position of mutation. (**D**). *SOST* model (AF-Q9BQB4-F1-model_v4, SWISS Model Repository) and focus on Gln24 substitution, in bold.

The first facial nerve palsy episode occurred at 13 months of age and was treated with steroids, resulting in partial recovery. In the following five months, three additional, always bilateral, episodes were observed unrelated to fever or other infectious events. Family history was remarkable for recurrent facial nerve palsy episodes in the paternal lineage, which were reported in four out of seven brothers of the patient’s father and in at least one first cousin of the proband (see Figure 2A).

At the time of the first clinical observation (age 14 months), neurological examination revealed only bilateral complete hyposthenia of mimic muscles, prevailing on the left side. Dysmorphological evaluation did not report dysmorphic features but only mild phenotypic variants such as slight asymmetry of the oral commissure, frontal bossing, wide forehead, thin hair, and apparently minimal nail dysplasia of the left-hand middle finger. No syndactyly was detected; weight and length were within the normal range according to age (Figure 1).

The sequence and timing of attainment of neurodevelopmental milestones were regular. Screening for major infections and autoimmune conditions was uninformative. Laboratory examinations for serum chemistry, thyroid hormones, calcium, phosphorus, parathormone, alkaline phosphatase, vitamins of complex B, and vitamin D were within normal limits.

Neurophysiology studies, including neurography, showed absence of cMAP (compound muscle action potential) evoked from the right facial nerve and the Blink Reflex test, while auditory brainstem responses (ABRs) and visual evoked potentials (VEPs) were normal. Ophthalmologic evaluation reported bilateral lagophthalmos. There were also unremarkable cardiologic assessments, with ECG, echocardiography, and whole abdomen ultrasound.

**Figure 3:**
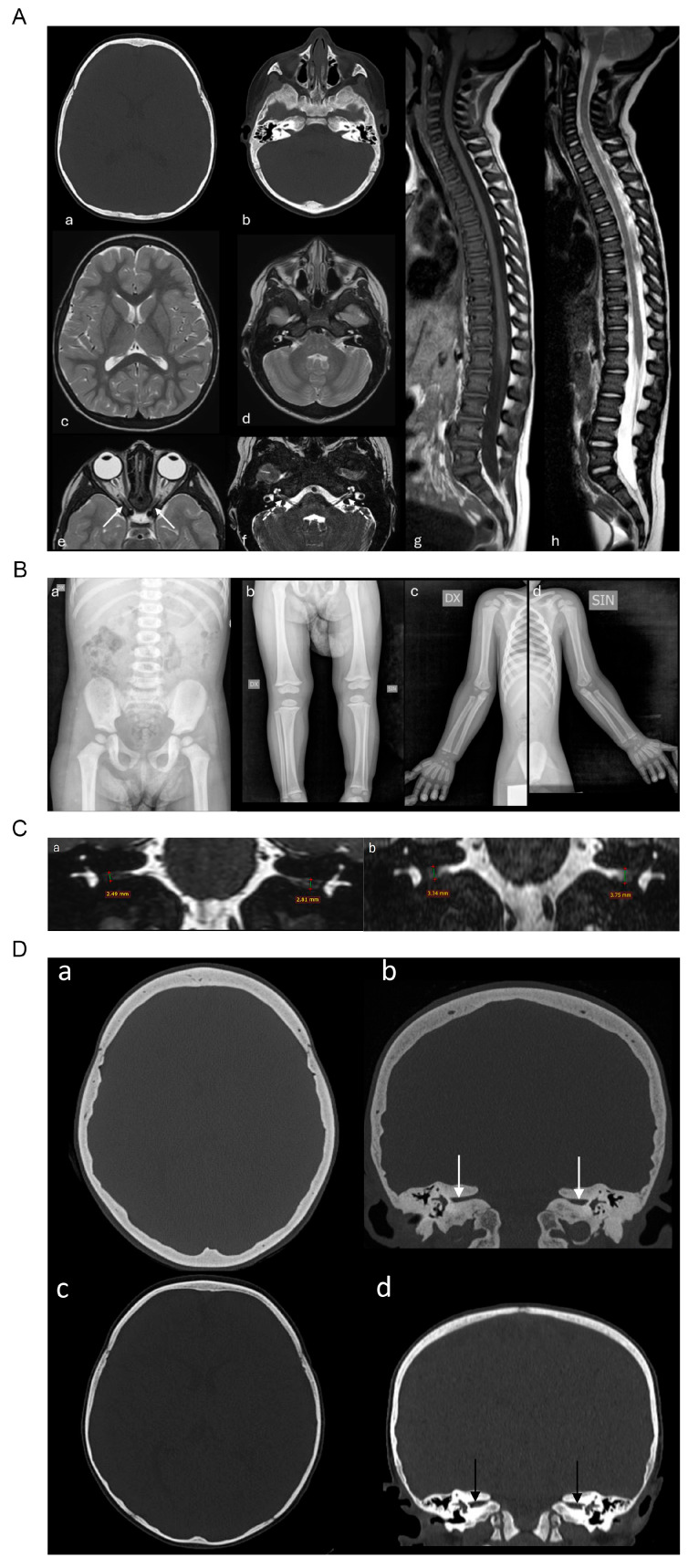
(**A**). Brain CT (**a**,**b**) and MRI (**c**,**d**) show sclerosis and hyperostosis with thickening of the cranial theca and basicranium. Arrows indicate the reduction in the caliber of optic foramina and internal acoustic canals (**e**,**f**). Spine MRI (**g**,**h**) reveals diffuse and mild sclerosis of vertebra both in T1W and T2W. (**B**). X-ray bone survey shows generalized increase in bone density with diffuse cortical thickening, showed in different sections (**a**,**b**,**c**,**d**). (**C**) Five-year follow-up coronal MRI with 3D-DRIVE sequences shows severe stenosis of internal acoustic canals (**b**) compared to baseline MRI (**a**). (**D**). Five-year follow-up CT (**a**,**b**) compared to baseline study (**c**,**d**) demonstrates progressive increase of hyperostosis and thickening of the cranial theca and temporal bones with severe stenosis of internal acoustic canals (arrows).

Baseline computed tomography (CT) scanning and magnetic resonance imaging (MRI) of the brain and the spinal cord showed suspicious signal of bilateral facial nerve hypoplasia and revealed hyperostosis with thickening of the cranial theca and of the trabecular basicranial bones with sclerosis of the cranio-cervical vertebral bodies, and reduction in the caliber of the internal acoustic canals and of the foramina of the optic nerves without alteration of their signal (Figure 3A). Total-body RX evaluation revealed a diffuse and slightly hyperdense aspect of all the bone segments examined (Figure 3B). Bone mineral density (BMD) measured by DEXA scan of the left femoris showed a BMD just above the range for the age (Z-score 102%, BMD 0.973 g/cm^3^).

In Figure 3C,D, comparison images of CT and MRI scanning are shown, displaying the progressive hyperostosis and thickening of the cranial theca and temporal bones with consequent advancement of the internal acoustic canals’ stenosis.

Today, the propositus is a 6-year-old girl with proportionate growth parameters within the upper normal range (stature cm 123 at 95 °ct; weight kg 23 at 75 °ct and head circumference cm 53 at 95 °ct) and no significant dysmorphic features except for what is already known from previous observations.

Over the years, she had about 2–3 recurrences of facial palsy per year, treated with oral steroids with partial recovery. The last neurological examination shows persistence of bilateral facial mimic musculature deficit without impairment of other cranial nerves. Instrumental investigations during follow-up showed stable neurophysiological findings (VEP and ABR). Tonal audiometry showed a normal hearing threshold, and the ocular fundus examination was also unaffected. However, neuroradiological investigations showed a progression of hyperostosis with an increasing reduction in the caliber of the optic foramina and internal acoustic canals during the years (see Figure 3D).

### 2.2. Genetic Analysis

Next-generation sequencing analysis using an in-silico panel focused on genes consistent with the clinical phenotype (Hyperostosis HP:0100774, facial nerve palsy, sclerotic skull base/vertebral endplates) was performed on DNA from the peripheral blood of both proband and parents.

NGS analysis and Sanger sequencing confirmation allowed us to identify a c.71delA (p.Gln24ArgfsTer33) homozygous nonsense variant in the first exon of the *SOST* gene [NM_025237.2] (see also Supplementary Table S1). This variant, not reported in ClinVAr, gnomAD, and dbSNP databases, is localized in the secretory domain, creates a premature stop codon, which determines the production of a truncated protein (Figure 2B,D). It is, therefore, classified as likely pathogenetic according to ACMG criteria. The variant has been identified in a homozygous state in the proband and in a heterozygous state in both parents’ DNA (Figure 2C), leading to the diagnosis of Sclerosteosis for the proband.

Further familiar segregation analysis showed the presence of the variant in other family members without a defined cosegregation in individuals who presented single or recurrent facial nerve paralysis (Figure 2A; see, for instance, individuals II-3 and III-5).

## 3. Discussion

The incidence of childhood peripheral facial nerve palsy is 5–21/100,000 [14,15,16]. Recurrent facial palsy is rare in the pediatric population and is reported in approximately 4.8% to 11% of cases [4,15,17,18,19,20]. In most patients with recurrent episodes, facial palsy is idiopathic; however, various infections, trauma, hypertension, chronic systemic diseases, and other syndromes, such as Moebius and Melkersson–Rosenthal, may also cause peripheral facial palsy [15,17,21,22,23]. In this work, we described a pediatric case of recurrent facial palsy secondary to hyperostosis of the skull and narrowing of the neural foramina in which acquired factors were excluded.

Given the patient’s age, family history and neuroradiological data, we performed exome sequencing, which revealed a novel homozygous variant c.71delA (p.Gln24ArgfsTer33) in the *SOST* gene, resulting in a frame shift-loss of function mutation.

*SOST* encodes for sclerostin, a protein expressed in several tissues but primarily secreted by osteocytes and, to lesser extent, osteoblasts, osteoclasts, mineralized hypertrophic chondrocytes, odontoblasts, and cementocytes [1]. Sclerostin functions as a negative regulator of bone formation by inhibiting the Wnt/β-catenin signaling pathway and, thus, the osteoblastic bone formation [2,3].

Osteoblast hyperactivation and bone overgrowth related to sclerostin deficiency are responsible for a group of metabolic bone disorders known as SOST-related sclerosing bone dysplasia.

To date, more than 100 individuals have been described with these disorders, most of them carrying loss-of-function mutation and showing a phenotype delineated as Sclerosteosis. The remaining patients are homozygous carriers of 52 kb deletion downstream of *SOST,* which removes a specific regulatory element, reduces the protein expression, and determines the phenotype known as Van Buchem Disease (VBD). Lastly, two different missense mutations at the same amino acid residues (the valine in position 21) have been associated with the SOST-related craniodiaphyseal dysplasia, the only dominant condition among the three related to *SOST* deficiency [4,10,11,12,13].

Thus, Sclerosteosis and Van Buchem Disease are the main, almost overlapping clinical phenotypes of SOST-related sclerosing bone dysplasia. Patients with Sclerosteosis, showing a harsher phenotype if compared to the VBD ones, display overgrowth of the skull bones and mandible, clavicles, ribs, pelvis, and long bones. Skull bone overgrowth might result in severe facial distortion, cranial nerves entrapment leading to deafness, visual impairment, and facial palsy [7].

Based on the available data from the literature, recurrent mono or bilateral facial palsy has been described in eight patients affected by Sclerosteosis with a median age of onset of 6 years (range 0.4–10 years) [12,24,25,26,27,28,29]), and at least six patients diagnosed with VBD showing recurrent facial palsy in pediatric age, four of them under 3 years [30,31].

Classically distinguishing features among the two conditions are the excessive linear overgrowth determining a final tall stature in Sclerosteosis but not in the VBD, the presence of hand abnormalities such as syndactyly, which is described in at least two third of the of the described Sclerosteosis patients but none of the VBD ones, and the high risk of increased intracranial pressure—one of the leading causes of morbidity and mortality—due to narrowing of the intracranial cavity, which is described in about 70% of individuals with Sclerosteosis but is a rare feature in individuals with Van Buchem Disease [4,7,9]

In our case, the proband has a linear growth within range, no syndactyly, and no facial distortion due to bone overgrowth. Her neuroradiological findings show diffuse sclerosis of the spongiosa bone of the skull base responsible for the entrapment of the optic, facial, and vestibulocochlear nerves; however, she only has facial palsies but no signs or symptoms of vision loss or hearing disorders. At the time of the last examination, no sign of intracranial hypertension was detected.

Even considering the limit of her young age and the hypothetical evolution of the clinical presentation, our patient seems to exhibit a phenotype closer to that of a VBD rather than that typical of Sclerosteosis.

To our knowledge, she is the first Italian case of *SOST*-related sclerosing bone dysplasia and the first Italian family carrying a novel loss-of-function variant in the *SOST* gene (Table S2, variant and related clinical phenotype).

Considering her mutation, the most N-terminal loss of function described so far, and its impact on protein structure, it would have been reasonable to expect a phenotype closer to typical Sclerosteosis.

We cannot exclude severe evolution over the years, but no major hand abnormalities and a linear growth within the normal range for age and sex let us discuss with the family about the hope of a milder-than-expected phenotype, specifying that only clinical evolution and close follow-up will define the phenotype and will allow a more adequate genotype–phenotype correlation.

In this respect, of interest was the peculiar anamnestic data about the significant recurrence of sporadic facial palsy episodes in several family members of the proband’s father. To date, to our knowledge, there is no report of correlation between the heterozygous state and the onset of isolated facial palsy episodes. The peculiar pathogenetic mechanism determining the palsies in the *SOST*-related sclerosing bone dysplasia (nerve entrapment due to narrowing of the neural foramina associated with the hyperostosis) and the absence of other hyperostosis-related symptoms make this link difficult to speculate. Only neuroimaging studies of the otherwise healthy individuals in the paternal lineage might highlight a putative association. Furthermore, no clear correlation between carriers of the mutation and facial palsy episodes can be established (see individuals II3 and III5 with a single facial palsy episode but no mutation or several other mutation carriers with no history of palsy).

## 4. Materials and Methods

### 4.1. Patients’ Enrollment

Patient and parents were enrolled at the Pediatric Neurology Unit of the Department of Neurosciences (Santobono-Pausilipon Children’s Hospital, Naples, Italy) during routine assessment for bilateral recurrent facial nerve palsy.

### 4.2. DNA Extraction, NGS Sequencing, and Bioinformatics Analysis

Genomic DNA was extracted using peripheral blood in EDTA according to the manufacturer’s instructions (MagCore Nucleic Acid Extraction Kit Diatech Pharmacogenetics, Ancona, Italy). DNA quantification was performed using a Qubit 3.0 Fluorometer with the Qubit dsDNA HS (High Sensitivity) Assay Kit fluorescent dye method. Following the manufacturer’s instructions, we used 100 ng of DNA to perform clinical exome library preparation (SeqCap EZ HyperCAp Library, Roche-SeqCap EZ MedExome Probes, Roche, Diagnostics, Monza, Italy). After the target region sequence was captured and enriched, the resulting DNA libraries were quantified with the Qubit dsDNA HS Assay Kit fluorescent dye method to determine equimolar amounts for each library. Sequencing was carried out on NovaSeq 6000 (Illumina Inc., San Diego, CA, USA) to a mean sequencing depth of at least 200×. Sequence data were aligned to the human reference genome GRCh37 (http://www.ncbi.nlm.nih.gov/projects/genome/assembly/grc/human/index.shtml, accessed on 11 October 2018) using the Burrows–Wheeler Aligner with default parameters.

Trimming, base calling, coverage analysis, and variant calling (vcf) were performed using an in-house bioinformatic pipeline taking advantage of the following free software: bcl to fastq version 2.20, Isaac Aligner version 4, GATK “Genome Analysis Toolkit” version 4, Samtools version 1.9, and Bedtools version 2.

Vcf analysis was performed using the Illumina Variant Interpreter filtered by quality > 15 and by small variant consequences such as stop gains, sand losses, splice donors, splice acceptors, splice region, frameshift indels, in-frame deletions, in-frame insertions, initiator codon (ATG) losses, missense protein altering, and incomplete terminal codon. Additional filtering was performed at a frequency < 0.05 in European populations using tools such as the 1000 Genomes Project (https://www.internationalgenome.org/ (accessed on 11 October 2018)), gnomAD (https://gnomad.broadinstitute.org/ (accessed on 11 October 2018)), Exome Aggregation Consortium (http://exac.broadinstitute.org/ (accessed on 11 October 2018), and the Human Gene Mutation Database (HGMD) (http://www.hgmd.cf.ac.uk/ac/ (accessed on 11 October 2018)).

A panel of genes related to phenotype (Hyperostosis HP:0100774) was analyzed to search for a causative variant. Variants were classified according to the American College of Medical Genetics and Genomics (ACMG) guidelines [32]. We only selected variants affecting coding exons or canonical splice sites. Finally, synonymous variants were filtered out to detect only rare variants (frequency of <0.1%) in both dbSNP138 and our in-house database containing >1000 exomes with high quality.

### 4.3. Sanger Sequencing Validation

The identified mutation was confirmed by Sanger sequencing in the proband, in the parents, and in the other family members. Polymerase chain reaction (PCR) was performed to amplify the fragments of suspected candidate mutation loci, and Sanger DNA sequencing validation was performed to detect the corresponding loci in the probands and family members participating in the study. Primer Premier 3 software (http://bioinfo.ut.ee/primer3-0.4.0 (accessed on 11 June 2020)) was used to design the target sequence primers 100 bp upstream and downstream from the mutation. Primer sequences are available on request. The genomic sequence from GenBank accession number NM_025237.2 was used as the reference sequence. PCR was performed to amplify the target fragments on a thermocycler instrument (SimpliAmp, ThermoFisher, Waltham, MA, USA), and the annealing temperature of PCR was 58 °C. DNA was amplified in a 50 µL reaction volume using AmpliTaq Gold 360 DNA polymerase (ThermoFisher), 0.2 µM each primer, and 100 ng of genomic DNA. The amplified products were isolated by electrophoresis on 1.5% agarose Genes 2021, 12, 1890 4 of 10 gel and purified using the QIamp Purification Kit (Qiagen, Valencia, CA, USA). Sequence analysis was performed on an ABI Prism 3500 genetic analyzer (ThermoFisher).

## 5. Conclusions

SOST-related sclerosing bone dysplasias are a group of rare diseases affecting bone growth, particularly cranial bones, and should be investigated in children with early onset of recurrent facial palsy, poorly responsive to common therapies. Furthermore, we would like to underline the importance of an early diagnosis that could allow proper monitoring and prevent/reduce severe neurological complications.

## Data Availability

Data is contained within the article and Supplementary Materials.

## Supplementary Materials

The following supporting information can be downloaded at: https://www.mdpi.com/article/10.3390/ijms26178175/s1.

## References

[1] Sebastian A., Loots G.G. (2017). Transcriptional Control of Sost in Bone. Bone.

[2] Sharifi M., Ereifej L., Lewiecki E.M. (2015). Sclerostin and Skeletal Health. Rev. Endocr. Metab. Disord..

[3] He W., Chen C., Pan C., Zhang M., Yu X., Wang D., Hu S. (2016). Sclerosteosis Caused by a Novel Nonsense Mutation of SOST in a Consanguineous Family. Clin. Genet..

[4] Appelman-Dijkstra N., Van Lierop A., Papapoulos S., Adam M.P., Feldman J., Mirzaa G.M., Pagon R.A., Wallace S.E., Amemiya A. (1993). SOST-Related Sclerosing Bone Dysplasias. GeneReviews^®^.

[5] Balemans W. (2001). Increased Bone Density in Sclerosteosis Is Due to the Deficiency of a Novel Secreted Protein (SOST). Hum. Mol. Genet..

[6] Brunkow M.E., Gardner J.C., Van Ness J., Paeper B.W., Kovacevich B.R., Proll S., Skonier J.E., Zhao L., Sabo P.J., Fu Y. (2001). Bone Dysplasia Sclerosteosis Results from Loss of the SOST Gene Product, a Novel Cystine Knot-Containing Protein. Am. J. Hum. Genet..

[7] Itin P.H., Keserü B., Hauser V. (2001). Syndactyly/Brachyphalangy and Nail Dysplasias as Marker Lesions for Sclerosteosis. Dermatology.

[8] Janssens K. (2002). Molecular Genetics of Too Much Bone. Hum. Mol. Genet..

[9] Sebastian A., Loots G.G. (2018). Genetics of Sost/SOST in Sclerosteosis and van Buchem Disease Animal Models. Metabolism.

[10] Loots G.G., Kneissel M., Keller H., Baptist M., Chang J., Collette N.M., Ovcharenko D., Plajzer-Frick I., Rubin E.M. (2005). Genomic Deletion of a Long-Range Bone Enhancer Misregulates Sclerostin in Van Buchem Disease. Genome Res..

[11] Balemans W. (2002). Identification of a 52 Kb Deletion Downstream of the SOST Gene in Patients with van Buchem Disease. J. Med. Genet..

[12] Kim S.J., Bieganski T., Sohn Y.B., Kozlowski K., Semënov M., Okamoto N., Kim C.H., Ko A.-R., Ahn G.H., Choi Y.-L. (2011). Identification of Signal Peptide Domain SOST Mutations in Autosomal Dominant Craniodiaphyseal Dysplasia. Hum. Genet..

[13] Bieganski T., Baranska D., Miastkowska I., Kobielski A., Gorska-Chrzastek M., Kozlowski K. (2007). A Boy with Severe Craniodiaphyseal Dysplasia and Apparently Normal Mother. Am. J. Med. Genet. Part A.

[14] Jenke A.C., Stoek L.-M., Zilbauer M., Wirth S., Borusiak P. (2011). Facial Palsy: Etiology, Outcome and Management in Children. Eur. J. Paediatr. Neurol..

[15] Karalok Z.S., Taskin B.D., Ozturk Z., Gurkas E., Koc T.B., Guven A. (2018). Childhood Peripheral Facial Palsy. Childs Nerv. Syst..

[16] Rowhani-Rahbar A., Baxter R., Rasgon B., Ray P., Black S., Klein J.O., Klein N.P. (2012). Epidemiologic and Clinical Features of Bell’s Palsy among Children in Northern California. Neuroepidemiology.

[17] Yılmaz Ü., Çubukçu D., Yılmaz T.S., Akıncı G., Özcan M., Güzel O. (2014). Peripheral Facial Palsy in Children. J. Child. Neurol..

[18] Wolfovitz A., Yehudai N., Luntz M. (2017). Prognostic Factors for Facial Nerve Palsy in a Pediatric Population: A Retrospective Study and Review. Laryngoscope.

[19] Chen W.-X., Wong V. (2005). Prognosis of Bell’s Palsy in Children—Analysis of 29 Cases. Brain Dev..

[20] Pitts D.B., Adour K.K., Hilsinger R.L. (1988). Recurrent Bell’s Palsy: Analysis of 140 Patients. Laryngoscope.

[21] Barr J.S., Katz K.A., Hazen A. (2011). Surgical Management of Facial Nerve Paralysis in the Pediatric Population. J. Pediatr. Surg..

[22] Drack F.D., Weissert M. (2013). Outcome of Peripheral Facial Palsy in Children—A Catamnestic Study. Eur. J. Paediatr. Neurol..

[23] Evans A.K., Licameli G., Brietzke S., Whittemore K., Kenna M. (2005). Pediatric Facial Nerve Paralysis: Patients, Management and Outcomes. Int. J. Pediatr. Otorhinolaryngol..

[24] Yagi H., Takagi M., Hasegawa Y., Kayserili H., Nishimura G. (2015). Sclerosteosis (Craniotubular Hyperostosis-Syndactyly) with Complex Hyperphalangy of the Index Finger. Pediatr. Radiol..

[25] Fayez A., Aglan M., Esmaiel N., El Zanaty T., Abdel Kader M., El Ruby M. (2015). A Novel Loss-of-Sclerostin Function Mutation in a First Egyptian Family with Sclerosteosis. BioMed Res. Int..

[26] Belkhribchia M.R., Collet C., Laplanche J.-L., Hassani R. (2014). Novel SOST Gene Mutation in a Sclerosteosis Patient from Morocco: A Case Report. Eur. J. Med. Genet..

[27] Magno De Andrade E., Beer-Furlan A., Duarte K., Fonoff E., Teixeira M. (2013). Management of Trigeminal Neuralgia in Sclerosteosis. Surg. Neurol. Int..

[28] Tholpady S., Dodd Z.H., Havlik R.J., Fulkerson D.H. (2014). Cranial Reconstruction for Treatment of Intracranial Hypertension from Sclerosteosis: Case-Based Update. World Neurosurg..

[29] Piters E., Culha C., Moester M., Van Bezooijen R., Adriaensen D., Mueller T., Weidauer S., Jennes K., de Freitas F., Löwik C. (2010). First Missense Mutation in the SOST Gene Causing Sclerosteosis by Loss of Sclerostin Function. Hum. Mutat..

[30] Van Egmond M.E., Dikkers F.G., Boot A.M., Van Lierop A.H.J.M., Papapoulos S.E., Brouwer O.F. (2012). A Rare Cause of Facial Nerve Palsy in Children: Hyperostosis Corticalis Generalisata (Van Buchem Disease). Three New Pediatric Cases and a Literature Review. Eur. J. Paediatr. Neurol..

[31] Dixon J.M., Cull R.E., Gamble P. (1982). Two Cases of Van Buchem’s Disease. J. Neurol. Neurosurg. Psychiatry.

[32] Richards S., Aziz N., Bale S., Bick D., Das S., Gastier-Foster J., Grody W.W., Hegde M., Lyon E., Spector E. (2015). Standards and Guidelines for the Interpretation of Sequence Variants: A Joint Consensus Recommendation of the American College of Medical Genetics and Genomics and the Association for Molecular Pathology. Genet. Med..

